# cfa-miR-143 Promotes Apoptosis via the p53 Pathway in Canine Influenza Virus H3N2-Infected Cells

**DOI:** 10.3390/v9120360

**Published:** 2017-11-25

**Authors:** Pei Zhou, Liqing Tu, Xi Lin, Xiangqi Hao, Qingxu Zheng, Weijie Zeng, Xin Zhang, Yun Zheng, Lifang Wang, Shoujun Li

**Affiliations:** 1 College of Veterinary Medicine, South China Agricultural University, Guangzhou 510642, China; zhoupei@scau.edu.cn (P.Z.); tulq0708@163.com (L.T.); l952963387@gmail.com (X.L.); haoxiangqi0829@163.com (X.H.); qingxu_z@126.com (Q.Z.); zengweijie765@163.com (W.Z.); xinzhangzhangxin@sina.com (X.Z.); emozy659@126.com (Y.Z.); wanglifangprince@163.com (L.W.); 2 Guangdong Provincial Key Laboratory of Prevention and Control for Severe Clinical Animal Diseases, Guangzhou 510642, China; 3 Guangdong Provincial Pet Engineering Technology Research Center, Guangzhou 510642, China

**Keywords:** cfa-miR-143, canine influenza virus, apoptosis, p53, caspase

## Abstract

MicroRNAs regulate multiple aspects of the host response to viral infection. This study verified that the expression of cfa-miR-143 was upregulated in vivo and in vitro by canine influenza virus (CIV) H3N2 infection. To understand the role of cfa-miR-143 in CIV-infected cells, the target gene of cfa-miR-143 was identified and assessed for correlations with proteins involved in the apoptosis pathway. A dual luciferase reporter assay showed that cfa-miR-143 targets insulin-like growth factor binding protein 5 (Igfbp5). Furthermore, a miRNA agomir and antagomir of cfa-miR-143 caused the downregulation and upregulation of Igfbp5, respectively, in CIV-infected madin-darby canine kidney (MDCK) cells. This study demonstrated that cfa-miR-143 stimulated p53 and caspase3 activation and induced apoptosis via the p53 pathway in CIV H3N2-infected cells. In conclusion, CIV H3N2 induced the upregulation of cfa-miR-143, which contributes to apoptosis via indirectly activating the p53-caspase3 pathway.

## 1. Introduction

MicroRNAs (miRNAs) are small, non-coding RNAs 18–22 nt in length that suppress gene expression via perfect or near-perfect base-pairing with complementary sequences in mRNA and participate in a wide range of biological processes [1,2]. Since 2002, researchers have explored the roles and relevant mechanisms of miRNAs in many diseases, such as cancer [3,4,5,6,7] and viral infections [8,9,10]. Moreover, miRNAs are considered potential biomarkers in some cancers based on miRNA expression analysis in cancerous tissues [11]. For example, high expression of miR-143 serves as a prognostic marker of gastric cancer [12]. miRNAs also have been recognized as potential antiviral and anticancer therapeutic micromolecules or targets [13,14].

Recently, miRNA expression profiles of influenza-infected hosts have been generated through high-throughput sequencing (HTS) and microarray technologies [15,16,17,18,19], suggesting that miRNAs play a crucial role in the pathology of influenza infection. miRNA expression is tissue-specific and dependent on the duration of time following virus exposure [20,21]. Therefore, specific miRNAs have been used to analyze antiviral effects. miR-150, miR-145 and miR-22 are differentially expressed in patients with severe H1N1 disease, while only miR-150 upregulation results in a decrease in the symptoms of H1N1 infection [22] via direct targeting of the Polymerase Basic Protein 2 (PB2) gene [10]. Influenza virus infection usually induces host inflammation and apoptosis [23,24], and miRNAs regulate cell growth and apoptosis pathways [25]. miR-136 is upregulated in H5N1-infected A549 cells and has shown potent antiviral activity [26]. Hsa-miR-145 was shown to be upregulated in human lung and trachea infected with 2009 pandemic H1N1 and inhibited viral replication by targeting the Hemagglutinin (HA) gene [10].

Since 2006, we have been committed to epidemic monitoring and exploring the pathogenesis of canine influenza virus (CIV). We have previously shown that cfa-miR-143 is upregulated in the lungs of CIV H3N2-infected dogs [27]. miR-143 regulates proliferation and apoptosis in cancers, including intestinal cancer, breast cancer, and gastric cancer [11,28,29]. It has been speculated that cfa-miR-143 directly binds to the 3′-untranslated region (UTR) of the insulin-like growth factor binding protein 5 gene (Igfbp5). Igfbp5 modulates the stimulatory effects of IGFs and is involved in cell growth and apoptotic processes in various cell types [30,31]. In human breast cancer cells, Igfbp5-induced apoptosis is associated with increased transcription of the proapoptotic regulator B-cell lymphoma-2 (bcl-2) associated X protein (bax) and decreased anti-apoptotic bcl-2 [32]. In this study, we explored the mechanism by which cfa-miR-143 targets Igfbp5 during apoptosis to enhance our understanding of the pathogenesis of CIV H3N2 infection.

## 2. Materials and Methods

### 2.1. Cells and Virus

Madin-Darby Canine Kidney (MDCK) cells (ATCC, CRL-2936) and 293T cells were cultured in Minimal Essential Medium (MEM) (Gibco, Waltham, MA, USA) and Dulbecco’s Modified Eagle Medium (DMEM) containing 5% fetal bovine serum (Gibco) and 200 IU penicillin-streptomycin (Gibco). Cells were harvested using 0.25% trypsin-EDTA (1×) (Gibco). A/Canine/Guangdong/01/2007(H3N2) was propagated in 11-day-old specific pathogen free (SPF) embryonated eggs. Viral titers were measured by determining the 50% tissue culture infectious dose (TCID_50_) according to the Reed-Muench calculation method in MDCK cells [33] and a plaque assay [34].

### 2.2. Infection

#### 2.2.1. Beagle Infections

One-month-old beagles were shown to be sero-negative by a hemagglutination-inhibition (HI) assay for seasonal influenza viruses (H1N1, H3N2, and influenza B virus) and for avian-origin CIV H3N2. All procedures and animal housing were carried out in the animal laboratory of South China Agricultural University. After the animals were lightly anesthetized using tiletamine–zolazepam (Virbac, Carros, France, 10–15 mg/kg), nine beagles were intranasally inoculated with 10^7^ EID_50_ (50% egg infectious dose)/animal of H3N2 CIV in a 1mL volume. For the uninfected controls, six additional beagles were inoculated with 1 mL of Phosphate Buffer Saline (PBS) and housed in a separate room. All animals, except for those euthanized for lung tissue collection, were monitored daily for body temperature and virus shedding for 14 days, and serum antibodies were detected at 14 days post-inoculation (dpi). At 3 dpi and 7 dpi, three animals in each inoculated group were euthanized. Lungs were collected to assess viral replication and miRNA expression. This study protocol was reviewed and approved by the Institutional Review Board of South China Agricultural University (identification code: 2014-07).

#### 2.2.2. Cell Infections

MDCK cells were inoculated with CIV H3N2 (Multiplicity Of Infection, MOI = 1.0) and incubated in 5% CO_2_ at 37 °C for 1 h. Then, the inoculum was discarded and replaced with MEM containing 2 μg/mL TPCK-trypsin and 0.2% bovine serum albumin (BSA), and the cells were incubated in 5% CO_2_ at 37 °C.

### 2.3. RNA Isolation

Small RNAs (<200 nt) were extracted from the lungs (*n* = 3) and MDCK cells using a miRNA extraction kit (Tiangen, Beijing, China). Total RNA was extracted from the same samples using Trizol reagent (Invitrogen, Carlsbad, CA, USA) according to manufacturer’s instructions. The total RNA concentration was measured by a spectrophotometer (Eppendorf, Hamburg, Germany) and either used for subsequent experiments or stored at −80 °C until use.

### 2.4. Real-Time Quantitative PCR (qPCR)

cfa-miR-143 levels were assessed with 10 ng of total miRNA from each sample in a TaqMan miRNA assay (has-miR143-3p) and TaqMan Universal Master Mix-II (Applied Biosystems, Foster City, CA, USA) as previously described [35]. A probe primer (U6 gene) (Applied Biosystems) was used to detect the internal control. Total RNA extracted using Trizol reagent was reverse-transcribed to cDNA with M-MLV reverse-transcriptase (TakaRa, Tokyo, Japan). Real-time qPCR was performed to analyze the transcriptional levels of Igfbp5, TP53 (tumor suppressor gene), bax (Bcl-2 associated X protein gene), bcl-2 (B-cell lymphoma/leukemia-2) and caspase3 using the primers (Invitrogen) listed in Table 1. All reactions were performed on a Light Cycler480 Real-time PCR system (Roche Molecular Systems, Basel, Switzerland). ΔΔCt values were calculated by normalizing threshold cycle (CT) values to U6 or GAPDH expression [36].

### 2.5. Luciferase Reporter Assay

The Igfbp5 gene (NCBI Reference Sequence: XM_847792.4) was predicted as the target gene of cfa-miR-143 based on biological information from the online software platforms miRDB and TargetScan-Vet. The genomic DNA of MDCK cells was extracted using a Genomic DNA Extraction Kit (Tiangen, Beijing, China), and the 3′-untranslated region (UTR) of the target gene was amplified using specific primers (Table 1). The PCR products were gel-purified, digested, and inserted into the digested psicheck-2 vector (Promega, Madison, WI, USA) between the *Xho*I and *Pme*I sites. Igfbp5-3′UTR target site mutations were introduced using specific primers (Table 1). cfa-miR-143 mimic and inhibitor were artificially synthesized (RiboBio, Guangzhou, China). Subsequently, psicheck2-Igfbp5 3′-UTR wild type (WT) or mutant (mut) was individually co-transfected into 293T and MDCK cells using Lipofectamine^®^ 2000 reagent (Invitrogen) with cfa-miR-143 mimic (50 nM) in 6-well plates. At 36 h post-transfection, luciferase activity was detected using a Dual-Glo Luciferase Assay Kit (Promega, Madison, WI, USA) according to the manufacturer’s protocol. Luminescence intensity was read using a GloMax® Discover System (GM3000, Promega, Madison, WI, USA). The relative fluorescence value was calculated from the ratio of renal luciferase to firefly luciferase activity. Transfections were performed in duplicate and repeated thrice [39].

### 2.6. Western Blotting

Total protein was extracted from MDCK cells infected with CIV H3N2 at specific time points using a Protein Assay Kit (KeyGen, Shanghai, China), and proteins including p53 (tumor suppressor protein), caspase3, cleaved caspase3, bax, bcl-2, PI3K (phosphatidylinositol-3 kinase), AKT, p-Akt and HA (hemagglutinin) were detected by Western blotting. The following primary antibodies were used: rabbit anti-Akt polyclonal antibody (Cell Signaling Technology, Danvers, MA, USA), rabbit anti-phosphorylated Akt (ser473, p-Akt) (Cell Signaling Technology), mouse anti-p53 monoclonal antibody (Beyotime, Shanghai, China), rabbit anti-caspase3 monoclonal antibody (Beyotime), rabbit anti-cleaved caspase3 (Asp175) monoclonal antibody (Beyotime), anti-bcl-2 monoclonal antibody (Bioss, Beijing, China), rabbit anti-bax monoclonal antibody (Bioss), mouse anti-PI3K p85(alpha) monoclonal antibody (ProteinTech, Wuhan, China), rabbit anti-phospho-PI3 Kinase p85 (Tyr458)/p55(Tyr199) antibody (Cell Signaling Technology), and a mouse anti-HA (H3N2 CIV) polyclonal antibody from our lab. The secondary antibodies were an IRDye 680RD goat anti-rabbit IgG (H + L) polyclonal antibody (LICOR) and IRDye 800CW goat anti-rabbit IgG (H + L) polyclonal antibody (LICOR) (Cell Signaling Technology). All antibody dilutions and the Western blotting procedure were performed per the manufacturer’s protocol. Relative expression levels were assessed relative to the reduced GAPDH protein expression detected using a mouse anti-GAPDH monoclonal antibody (ZSGB-bio, Beijing, China).

### 2.7. Flow Cytometry

For flow cytometry, cells were harvested by 0.25% trypsin (no EDTA), followed by washing with PBS. Cell death was assessed by two-color flow cytometric analysis using an Annexin-APC/PI apoptosis kit (Ebioscience) (FC500, Beckman, Brea, CA, USA).

### 2.8. Statistical Analysis

Statistical significance was determined using the conventional Student’s *t* test. All assays were run in triplicate. The data are shown as the mean ± S.E., and a *p* value < 0.05 was considered statistically significant (* *p* < 0.05; ** *p* < 0.01; *** *p* < 0.001).

## 3. Results

### 3.1. Experimental Infection with CIV H3N2 in Dogs

All dogs inoculated with virus showed influenza-like symptoms and elevated temperature 3 days after inoculation (Figure S1A). Virus shedding was detected from 1 to 8 dpi from nasal swabs (Figure S1B). Viral replication in the lungs was detected at 0 dpi, 3 dpi and 7 dpi, and the mean viral titers of these time points were 0.00, 5.75, and 2.50 logTCID_50_/mL, respectively (Figure S1C). Seroconversions were detected at 14 dpi.

### 3.2. cfa-miR-143 Upregulation with CIV H3N2 Infection

**Figure 1:**
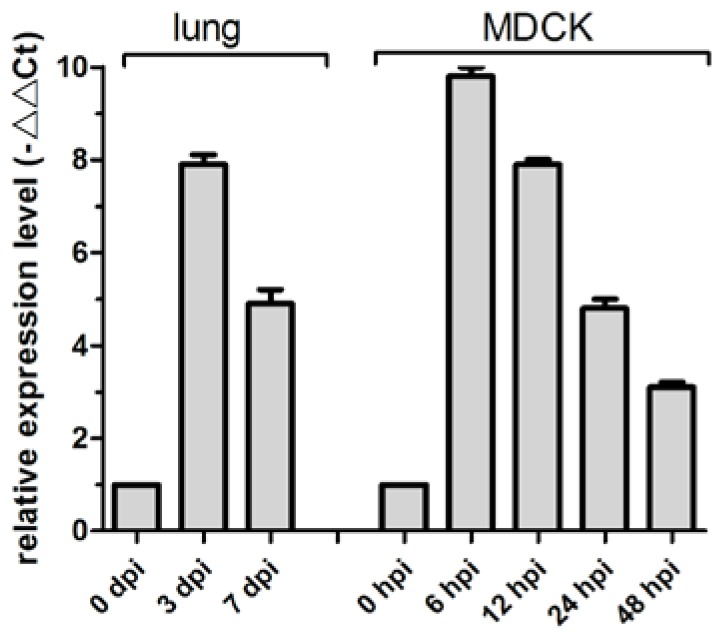
cfa-miR-143 expression was measured in canine influenza virus (CIV)-infected lungs and Madin-Darby Canine Kidney (MDCK) cells by real-time qPCR. The relative expression is depicted relative to the negative control at each time point. hpi: hour post-inoculation.

MiR-143 regulates proliferation and apoptosis in cancers, including intestinal cancer, breast cancer, and gastric cancer [11,28,29]. Previously, we determined that cfa-miR-143 is upregulated in CIV H3N2-infected dog lungs in a deep sequencing study [27]. In the current study, cfa-miR-143 expression in CIV-infected lungs increased by 7.9-fold at 3 dpi and by 4.9-fold at 7 dpi compared to that in uninfected dogs (0 dpi) (Figure 1). The significantly increased expression of cfa-miR-143 was also observed in CIV-infected MDCK cells at different time points.

### 3.3. cfa-miR-143 Targets the Igfbp5 Gene

**Figure 2:**
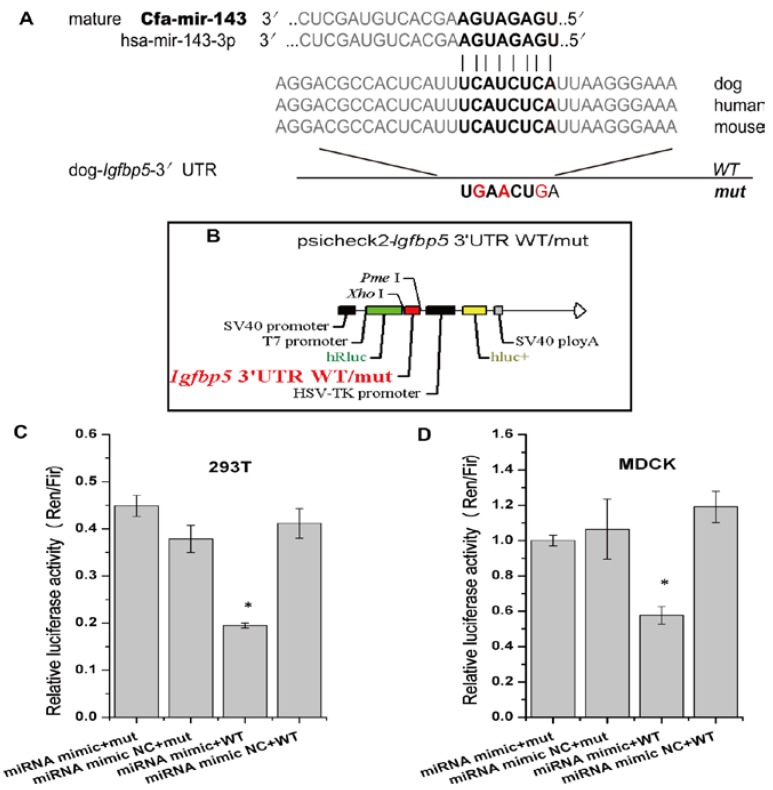
cfa-miR-143 targets the Igfbp5 gene. (**A**) A mutation in the 3′-UTR of the Igfbp5 gene was designed in a seed sequence site for binding with cfa-miR-143. The inserted mutation is marked (red); (**B**) The WT (wildtype) and mut (mutation) genes were inserted into a dual fluorescence reporter retroviral vector (psicheck-2). The insertion was ligated between the human Renilla luciferase (hRlu) gene and the promoter of the human firefly luciferase (hFlu) gene. The recombined plasmids, psicheck2-Igfbp5 3′UTR WT and psicheck2-Igfbp5 3′UTR mut, were transfected into 293T and MDCK cells along with cfa-miR-143 mimic or mimic NC (Negative Control); (**C**,**D**) Detection of relative luciferase activity showed that the hRlu/hFlu ratio was significantly lower (*p* < 0.05) in cells co-transfected with mimic and WT than in those with mimic and mut in both 293T and MDCK cells. Differences were considered statistically significant at *p* < 0.05. HSV-TK: herpesvirus thymidine kinase. Differences in expression levels were considered significant if *p* < 0.05 (* *p* < 0.05).

The online biological software platforms miRDB and TargetScan-Vet were used to predict target genes. The Igfbp5 gene, which is related to cell apoptosis genes, is the unique predicted target of cfa-miR-143 in apoptosis pathway (Figure 2A). Subsequently, we obtained the dog Igfbp5 3′-UTR gene and inserted a mutation into the gene (Figure 2A, Bold). WT and mut Igfbp5 3′-UTR gene sequences were inserted into a dual luciferase reporter retroviral vector (psicheck-2) (Figure 2B). The recombined psicheck2-Igfbp5 3′-UTR WT/mut plasmids were separately co-transfected into MDCK and 293T cells along with cfa-miR-143 mimic or mimic NC (negative control). After 36 h, the relative luciferase activity of cells co-transfected with the WT plasmid and miRNA mimic was significantly lower than that of cells co-transfected with the mut plasmid and mimic or the WT plasmid and mimic NC (Figure 2C,D). This result suggested that the cfa-miR-143 mimic suppressed transcription of the Igfbp5 gene by binding to the 3′-UTR-Igfbp5.

### 3.4. CIV H3N2 Triggers Apoptosis

**Figure 3:**
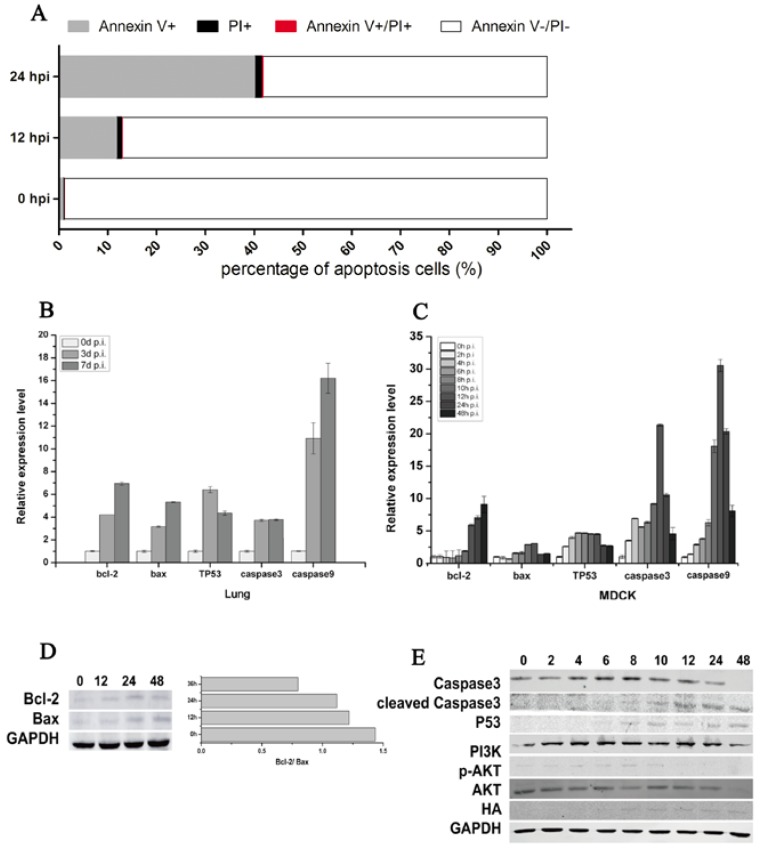
CIV H3N2-induced apoptosis. Cell apoptosis was assessed using an Annexin-APC/PI (Propidium Iodide) apoptosis kit for flow cytometry (**A**); Lungs and cells were collected to isolate RNA at specific time points. Real-time qPCR was performed to detect the expression of the apoptosis-associated genes bcl-2, bax, TP53, caspase3, and caspase9, and relative expression levels are shown according to the ratio of the infected and uninfected groups at each time point (**B**,**C**); In addition, apoptosis-associated proteins were detected by Western blotting (**D**,**E**).

**Figure 4:**
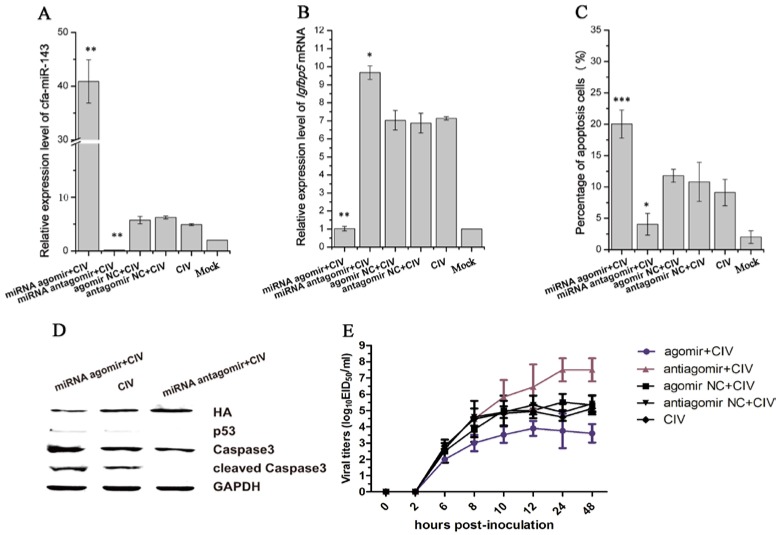
Detection of apoptosis and virus titers after pre-transfecting with agomir and antagomir in CIV-infected MDCK cells. First, Madin-Darby Canine Kidney (MDCK)cells were transfected with agomir and antagomir and cultured for 36 h. The cells were then infected with CIV (Multiplicity Of Infection, MOI = 1.0). cfa-miR-143 levels were significantly upregulated in cells transfected with miRNA agomir (**A**); cfa-miR-143 agomir and antagomir significantly restricted and enhanced the expression of Igfbp5 mRNA, respectively, compared with the expression of untreated CIV-infected MDCK cells (**B**); After 12 hpi, the cell supernatants were discarded and washed with phosphate buffer, and numbers of apoptotic cells were subsequently detected by flow cytometry. Upregulation of cfa-miR-143 by agomir significantly increased apoptosis, and downregulation by antagomir increased inhibited apoptosis (**C**); In addition, Western blotting was carried out to detect apoptosis-associated proteins. The upregulation of cfa-miR-143 by agomir inhibited HA expression, and enhanced p53, caspase3 and cleaved caspase3 protein expression. Inhibition of cfa-miR-143 by the antagomir caused the opposite effect (**D**); at 0, 2, 6, 8, 10, 12, 24, 48 hpi, virus was collected and titers were calculated by EID50 (**E**). Differences in expression levels were considered significant if *p* < 0.05 (* *p* < 0.05; ** *p* < 0.01; *** *p* < 0.001).

Flow cytometric analysis showed that CIV-induced MDCK cell apoptosis increased from 12.88% at 12 hpi to 41.74% at 24 hpi (Figure 3A). The percent of Annexin V+ shed was 12.10% at 12 hpi and 41.00% at 24 hpi, which indicated that CIV H3N2 induced the early apoptosis of MDCK cells. To further explore the mechanism of early cell apoptosis, apoptosis signaling gene expression was detected by qPCR and WB. The mRNA relative expression of *bcl-2*, *bax*, *TP53*, *caspase3* and *caspase9* increased in virus-infected lungs (Figure 3B) and in virus-infected MDCK cells (Figure 3C). The protein expression of HA appeared at 8 hpi and then maintained a stable expression level (Figure 3E), which consistent with the virus titers (Figure 4E) indicated that CIV H3N2 replicated in the cells and maintained stable virus titers. The protein expression of bcl-2 and bax increased in infected MDCK cells (Figure 3D), and the expression rate of bcl-2 and bax (bcl-2/bax) gradually increased from 0.8 at 12 hpi to 1.4 at 48 hpi (Figure 3D). The protein expression of caspase3 increased from 0 hpi to 8 hpi, and cleaved caspase3 significantly increased from 8 hpi to 48 hpi, which indicated cell apoptosis was occurring. The PI3K/AKT pathway has been shown to be activated in influenza virus-infected MDCK cells through the NS1 protein [40]. In this study, Akt phosphorylation (p-Akt) occurred during early infection (before 10 hpi) (Figure 3E), which is the viral replication stage [41,42], indicating that cell apoptosis was inhibited during the viral replication stage. The protein expression of p53 gradually increased from 6 hpi to 48 hpi (Figure 3E), suggesting that cell apoptosis was induced by the p53 pathway.

### 3.5. cfa-miR-143 Induces Apoptosis via the p53 Pathway in CIV-Infected MDCK Cells

cfa-miR-143 is significantly upregulated with CIV H3N2 infection and effectively targets the Igfbp5 gene. Thus, the effects of cfa-miR-143 upregulation on CIV-infected cells were explored in this study. The optimal concentrations of agomir and antagomir were determined by assessing cfa-miR-143 expression by qPCR. One hundred nanomolar agomir and 100 nM antagomir significantly stimulated and inhibited cfa-miR-143 expression, respectively (Figure S2A,B). Therefore, 100 nM agomir and 100 nM antagomir were used as optimal concentrations in all downstream experiments.

cfa-miR-143 expression significantly increased with cfa-miR-143 agomir transfection and significantly decreased with cfa-miR-143 antagomir transfection (Figure 4A). The expression of the target gene Igfbp5 significantly increased with cfa-miR-143 antagomir transfection in CIV-infected cells (Figure 4B). However, Igfbp5 expression significantly decreased with cfa-miR-143 agomir transfection in CIV H3N2-infected cells (Figure 4B). Meanwhile, the miRNA agomir significantly increased the percentage of apoptotic cells (Figure 4C) by activating p53 and caspase3 in CIV-infected cells (Figure 4D). Interesting, virus production decreased with miRNA agomir transfection and increased with miRNA antagomir transfection (Figure 4E).

**Figure 5:**
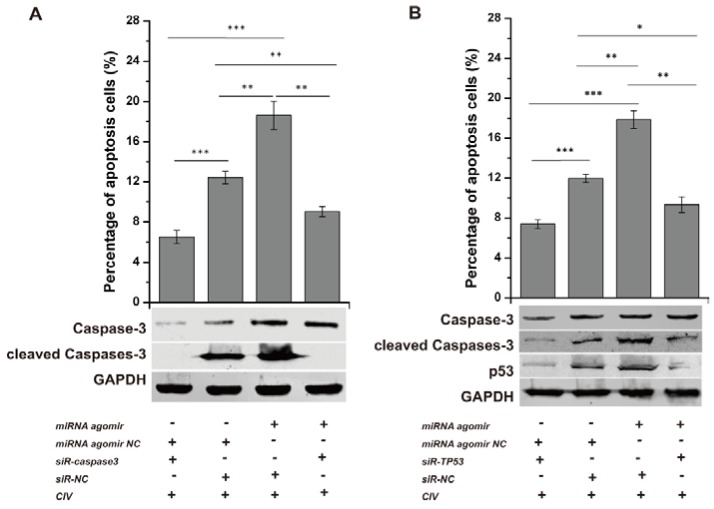
Interference assay. siRNAs were transfected into Madin-Darby Canine Kidney (MDCK) cells for 36 h and subsequently infected by CIV H3N2 (Multiplicity Of Infection, MOI = 1.0) at 12 h. The cells were collected to detect apoptosis. To explore the function of the p53 pathway in CIV-infected MDCK cells, siRNAs targeting TP53 (siR-TP53) and caspase3 (siR-caspase3) were applied individually or jointly with agomir and antagomir. siR-caspase3 restricted the expression and activation of caspase3 as shown by Western blotting and decreased the apoptosis induced by the cfa-miR-143 agomir as shown by flow cytometry (**A**); siR-TP53 restricted the expression and activation of p53 and the activation of caspase3 as shown by Western blotting and decreased apoptosis induced by cfa-miR-143 agomir as shown by flow cytometry (**B**). Differences in expression levels were considered significant if *p* < 0.05 (* *p* < 0.05; ** *p* < 0.01; *** *p* < 0.001).

Conversely, the miRNA agomir and siR-TP53 or siR-caspase3 (sequences shown in Table 2) were co-transfected into MDCK cells, followed by CIV H3N2 infection at 12 h post-transfection (hpt). The optimal concentrations of siRNA were determined by assessing the expression of their target genes by qPCR. All concentrations (25 nM, 50 nM, 100 nM) of siR-TP53 and siR-caspase3 inhibited the expression of TP53 and caspase3, respectively (Figure S2C). p53 expression levels and caspase3 activation were determined by WB, and the number of apoptotic cells was detected by flow cytometry. Cell apoptosis was significantly reduced with p53 and caspase3 interference (Figure 5A,B: column 1, column 2), suggesting that CIV-induced apoptosis is likely a p53-dependent pathway activating the downstream apoptotic factor caspase3. p53 levels and caspase3 activation increased with co-transfection of the miRNA agomir and siR-NC and were reduced with the miRNA agomir and siR-TP53 or siR-caspase3 co-transfection (Figure 5A,B: lane 3, lane 4). Additionally, apoptotic cells increased from 12.43 ± 0.64% to 18.62 ± 1.41% (*p* < 0.01) due to the action of miRNA agomir but decreased to 9.04 ± 0.51% (*p* < 0.01) with siR-caspase3 (Figure 5A: column 3, column 4) and decreased from 17.87 ± 0.88% to 9.33 ± 0.79% (*p* < 0.01) with siR-TP53 (Figure 5B: column 3, column 4). These results indicated that miRNA agomir-induced apoptosis was inhibited via interference with p53 and subsequently restricted activation of the downstream effector caspase3.

## 4. Discussion

Influenza virus binds to the cell surface and then gains entry via endocytosis, which activates various cellular responses, including miRNAs that act as host defense mechanisms against viral infection. For example, host miR-let-7c inhibits H1N1 M1 protein expression to protect lung epithelial cells from viral damage [43]. In our previous study, we found that miR-143 expression was upregulated at 4 dpi in the CIV-infected beagle lung [27]. Many studies have demonstrated that miR-143 regulates growth [44], proliferation [45] and apoptosis [46] in cells. In the present study, we explored the functions of cfa-miR-143 in CIV-infected MDCK cells. We observed the varying expression of cfa-miR-143 in vivo and in vitro during a CIV H3N2 infection time course. miRNA enrichment was accompanied by a wide range of innate immune factors that are regarded as potential key regulators of the immune response to viral infection [47]. Therefore, the fluctuating expression cfa-miR-143, which was upregulated during the early stage and then gradually decreased, suggests that it plays a role in both the antiviral innate immune response and adaptive immune response [48].

miRNAs are known to target specific genes and regulate their expression [45]. Igfbp5 is the unique target gene of cfa-miR-143 in apoptosis pathway. As a critical member of the IGF axis, Igfbp5 has an important role in controlling cell survival, differentiation, and apoptosis by restricting IGF binding to the IGF1 receptor [49]. We demonstrated that the 3′-UTR of Igfbp5 mRNA is a direct target of miR-143 in a dual luciferase experiment. Furthermore, upregulating and downregulating cfa-miR-143 levels by pre-transfecting miRNA agomir/antagomir in MDCK cells showed that cfa-miR-143 directly controls Igfbp5 expression at the transcriptional level. A negative correlation was found between the rate of apoptosis and Ifgbp5 levels in CIV-infected MDCK cells (Figure 4B,C), suggesting that there is potential connection between Ifgbp5 inhibition and apoptosis increased. The specific mechanism need be further explored.

Apoptosis is programmed cell death, which has been demonstrated in hosts with influenza A virus infection [23,50]. A previous study showed that CIV H3N2, a common epidemic influenza subtype in dogs, also stimulates the expression of genes related to inflammation and apoptosis in the dog lung [24]. A reduction in cell cycle progression and increased apoptosis were observed upon miR-143 over-expression by targeting DNMTA3A mRNA in human leukemia cells [51]. Cell proliferation and apoptosis increased with miR-143 over-expression in colorectal cancer cells [46]. Igfbp5, the target gene of cfa-mir-143, plays roles in the regulation of cellular senescence via a p53-dependent pathway and in aging-associated vascular diseases [52]. Therefore, cfa-miR-143 may be involved in p53-dependent apoptosis in CIV-infected MDCK cells by targeting Igfbp5. Influenza virus-inducible apoptosis pathways primarily include the cell-membrane death receptor extrinsic pathway and the mitochondrial intrinsic pathway [53]. Other studies have suggested that programmed cell death and apoptosis are either largely caspase-dependent or independent [54]. In this study, real-time qPCR analysis showed that upregulation of most apoptosis-associated genes activated multiple apoptosis pathways. Moreover, the increased expression of caspase-family mRNAs suggested activation of the caspase3-dependent apoptosis pathway in the CIV-infected host (lung and MDCK cells). p53 expression was persistently increased after virus infection in MDCK cells, which is consistent with the relatively steady expression of TP53 mRNA levels, suggesting the upregulation of p53 transcription at the same site as influenza infection [55]. Upregulation of the caspase3 and cleaved caspase3 proteins suggests the involvement of caspase3 in apoptosis [56].

Manikandan et al. indicated that miR-143 likely indirectly upregulates p53 in oral squamous cell carcinoma [57]. To understand the role and significance of p53 and its relationship with cfa-miR-143 in CIV-induced apoptosis, siRNA assays were performed. The data showed that cfa-miR-143 upregulated p53 expression levels, resulting in enhanced apoptosis in CIV-infected MDCK cells. Interferon regulatory 3 (IFR3), stimulated by influenza virus, is known to trigger activation of the p53-dependent apoptosis pathway [58]. Furthermore, p53 targets the downstream type I interferon-induced genes, such as interferon regulatory 5 (IRF5) and interferon stimulated gene 15 (ISG5), and may contribute to the antiviral cellular response and prevent viral replication [55,59,60]. p53 expression was increased by the cfa-miR-143 agomir and drastically accelerated by caspase-3 activation, sequentially resulting in increased apoptosis. The activation of caspase3 as an effector of apoptosis plays a crucial role in influenza virus propagation and the induction of apoptosis, as previously described [61]. p53-mediated Fas expression is conducive to the activation of the downstream effector caspase3/7, triggering tumor cell apoptosis as a self-protective program against virus infection [62]. Restricting caspase3 expression with siRNA significantly lowered the expression and activation of caspase3, reducing the effects of the cfa-miR-143 agomir and inhibiting apoptosis. This result indicated that the activation of caspase3 is essential for CIV-induced apoptosis. As an apoptosis effector, caspase3 is activated by multiple pathways (e.g., the p53 signaling pathway, PI3K/AKT signaling pathway and Ca^2+^-induced pathway).

## 5. Conclusions

CIV H3N2 induced the upregulation of cfa-miR-143, which promotes cell apoptosis by indirectly activating the p53-caspase3 pathway.

## Figures and Tables

**Table 1:** The following primers were used in our study.

Primer Name	Primer Sequence	References
Igfbp5 (WT)-Forward	5′-CTCGAGGTCCTCCCCTCGCCCCATCCCATCC-3′	NCBI Reference Sequence: XM_847792.4
Igfbp5 (WT)-Reverse	5′-GTTTAAACTAAATGAGATGAAATGAGTGGCGTC-3′	
Igfbp5 (mut)-Forward	5′-CATTTGAACTGATTTAGT-3′ (5′ phosphorylation)	
Igfbp5 (mut)-Forward	5′-CAGTTCAAATGAGTGGCGTCCT-3′ (5′ phosphorylation)	
Igfbp5-Forward (qPCR)	5′-GACTCCAGCCAGCACCT-3′	
Igfbp5-Reverse (qPCR)	5′-AGACCTTGCTAGCGATTCCGA-3′	
TP53-Forward	5′-ACAGTAGTGACGGTCTTGCC-3′	GenBank: AB020761.1
TP53-Reverse	5′-CAACCTCGGGTGGCTCATAA-3′	
bcl-2-Forward	5′-CATGCCAAGAGGGAAACACCAGAA-3′	[37]
bcl-2-Reverse	5′-GTGCTTTGCATTCTTGGATGAGGG-3′	
bax-Forward	5′-TTCCGAGTGGCAGCTGAGATGTTT-3′	
bax-Reverse	5′-TGCTGGCAAAGTAGAAGAGGGCAA-3′	
caspase3-Forward	5′-TTCATTATTCAGGCCTGCCGAGG-3′	
caspase3-Reverse	5′-TTCTGACAGGCCATGTCATCCTCA-3′	
caspase8-Forward	5′-ACAAGGGCATCATCTATGGCTCTGA-3′	
caspase8-Reverse	5′-CCAGTGAAGTAAGAGGTCAGCTCAT-3′	
caspase9-Forward	5′-TCAGTGACGTCTGTGTTCAGGAGA-3′	
caspase9-Reverse	5′-TTGTTGATGATGAGGCAGTAGCCG-3′	
GAPDH-Forward	5′-TTCCACGGCACAGTCAAG-3′	[38]
GAPDH-Reverse	5′-ACTCAGCACCAGCATCAC-3′	

WT: wildtype; mut: mutation; GAPDH: glyceraldehyde-3-phosphate dehydrogenase.

**Table 2:** The siRNA sequences were artificially synthetized accordingly.

siRNA Name	Strand	Sequence
siR-caspase3	positive-sense strand	5′ GCAGCAAACCUCAGGGAAA dTdT 3′
siR-caspase3	antisense strand	3′ dTdT CGUCGUUUGGAGUCCCUUU 5′
siR-TP53	positive-sense strand	5′ CCAUCCACUACAACUACAU dTdT 3′
siR-TP53	antisense strand	3′ dTdT GGUAGGUGAUGUUGAUGUA 5′

## Supplementary Materials

The following are available online at http://www.mdpi.com/1999-4915/9/12/360/s1. Figure S1: The body temperature (A), virus shedding (B) and viral replication in lungs (C) of dogs with CIV H3N2 infection. Figure S2: The optimal concentrations of agomir, antagomir and siRNA by assessing the expression of their target genes. The stimulation and inhibition of the cfa-miR-143 expression by agomir (A) and antagomir (B), respectively. The knockdown of the TP-53 and caspase3 by siR-TP53 and siR-caspase3, respectively (C).
