# Supplementary figures and images for: cfa-miR-143 Promotes Apoptosis via the p53 Pathway in Canine Influenza Virus H3N2-Infected Cells
Source: Viruses. 2017 Nov 25;9(12):360. doi: 10.3390/v9120360 (PMC5744135; doi:10.3390/v9120360)

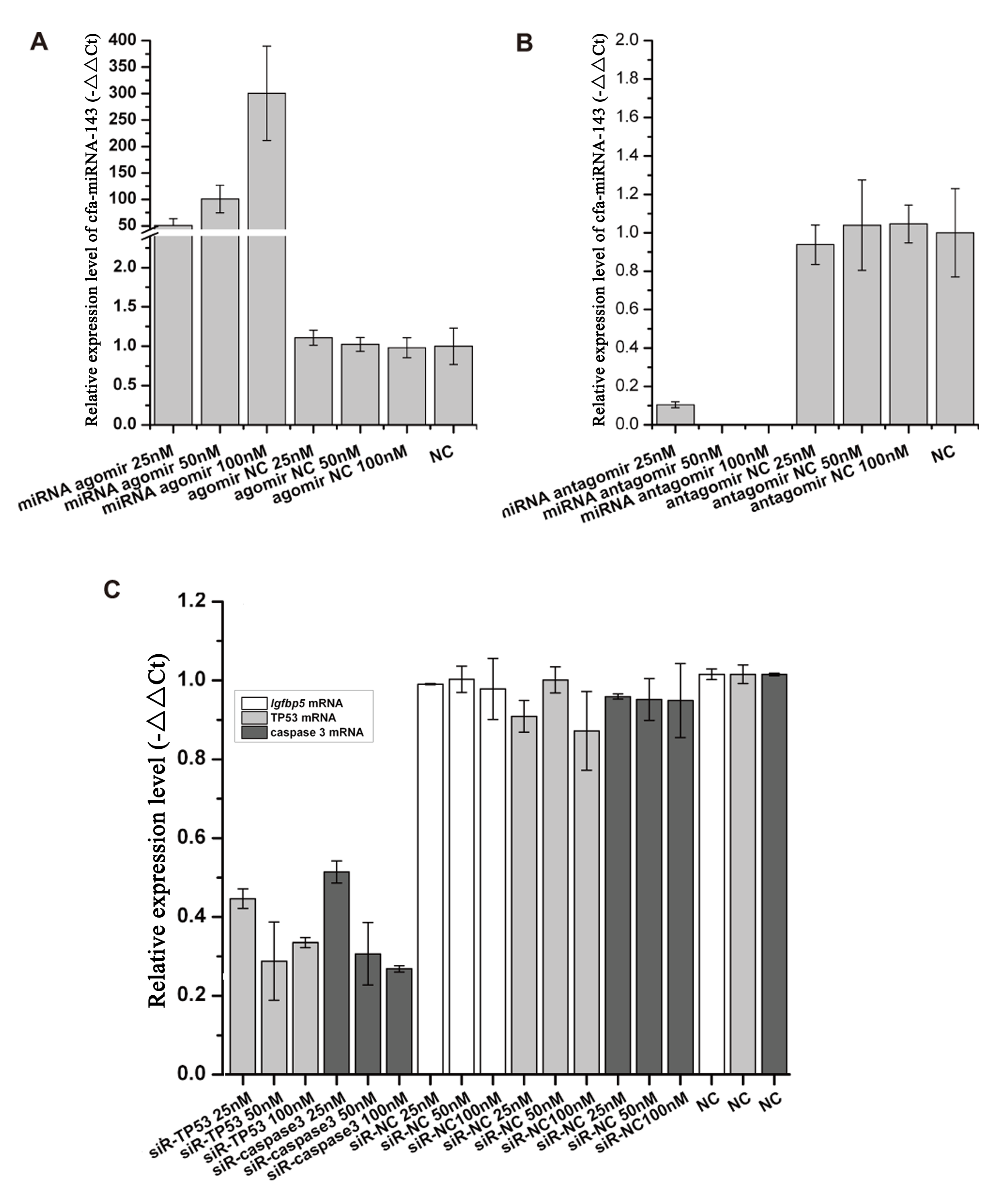

Supplement: Supplementary file 1 [file viruses-09-00360-s001.zip › FigureS2.tif]

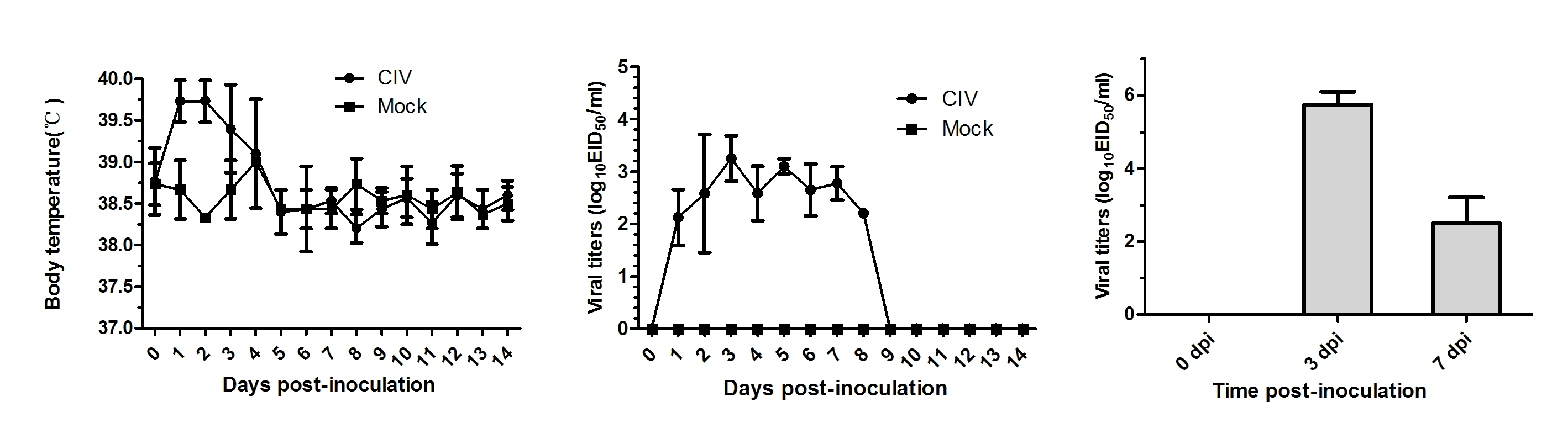

Supplement: Supplementary file 1 [file viruses-09-00360-s001.zip › FigureS1.tif]
